# Adiponectin exacerbates collagen-induced arthritis via enhancing Th17 response and prompting RANKL expression

**DOI:** 10.1038/srep11296

**Published:** 2015-06-11

**Authors:** Xiaoxuan Sun, Xiaoke Feng, Wenfeng Tan, Na Lin, Minhui Hua, Yu Wei, Fang Wang, Ningli Li, Miaojia Zhang

**Affiliations:** 1 Department of Rheumatology and Immunology, The First Affiliated Hospital of Nanjing Medical University, Jiangsu, China.; 2 Department of Traditional Chinese Medicine, The First Affiliated Hospital of Nanjing Medical University, Jiangsu, China.; 3 Department of Cardiology, The First Affiliated Hospital of Nanjing Medical University, Jiangsu, China; 4 Shanghai Institute of Immunology, Institute of Medical Sciences, Shanghai Jiao Tong University School of Medicine, Shanghai, China

## Abstract

We previously reported adiponectin (AD) is highly expressed in the inflamed synovial joint tissue and correlates closely with progressive bone erosion in Rheumatoid arthritis (RA) patients. Here, we investigate the role of adiponectin in regulating Th17 response and the expression of receptor activator of nuclear factor-κB ligand (RANKL) in mice with CIA mice by intraarticularly injection of adiponectin into knee joints on day 17, day 20 and day 23 post first collagen immunization. The increased adiponectin expression was found in inflamed joint tissue of collagen-induced arthritis (CIA) mice. Adiponectin injection resulted in an earlier onset of arthritis, an aggravated arthritic progression, more severe synovial hyperplasia, bone erosion and osteoporosis in CIA mice. CD4^+^IL-17^+^ Th17 cells, IL-17 mRNA and RANKL mRNA expression were markedly increased in the joint tissue of adiponectin treated CIA mice. Moreover, adiponectin treatment markedly enhanced Th17 cell generation from naive CD4^+^ T cells ***in vitro***, which accompanied by the high expression of Th17 transcription factor ROR-γt, and Th17 cytokine genes included IL-22 and IL-23. This study reveals a novel effect of adiponectin in exacerbating CIA progression by enhancing Th17 cell response and RANKL expression.

Rheumatoid arthritis (RA) is a common rheumatic disease that is characterized by chronic inflammation, joint destruction and progressive disability^1^. Despite intensive efforts in the development of new therapies to prevent the disease progress in RA^2^, a proportion of patients still failed to current synthetic or biological disease-modifying anti-rheumatic drugs (DMARDs) therapy^3^. Therefore, it is still a critical challenge to find novel targets for RA therapy.

Although the precise etiology of RA still remains elusive^1^, substantial evidence has suggested that T cells, B cells and the complex interaction of multiple pro-inflammatory cytokines, including tumor necrosis factor-α (TNF-α), interleukin (IL)-6, IL-1 and IL-17, play a critical role in the pathophysiology of RA^4^,^5^. Therapies targeting against TNF-α, IL-1 and IL-6 have demonstrated the favorable clinical outcomes in patients with RA, highlighting the critical role of pro-inflammatory cytokines in RA pathophysiology.

Adipose tissue or the adipocytes in joints have long been considered as none-bioactive cells and only devoted to energy storage. During recent years, there is growing evidence that adipokines produced by white adipose tissue including adiponectin (AD), leptin, visfatin and resistin play an important role in regulating immune and inflammatory processes^6^,^7^. AD is the most abundant adipokine, being present at concentrations of 5–30 μg/ml in circulation. It is produced prevalently by adipose tissues, but is also secreted by skeletal muscles, cardiacmyocytes, and endothelial cells. AD exerts its functions by acting on its receptors: adiponectin receptor 1 (AdipoR1) and adiponectin receptor 2 (AdipoR2)^8^.

Our previous studies have demonstrated that AD is highly expressed in the inflamed synovial joint tissue and correlates closely with progressive bone erosion in RA patients^9^. Moreover, local levels of AD are positively correlated with IL-6 in RA synovial fluids. Stimulation of RA synoviocytes with AD could trigger a high expression of IL-6 and monocyte chemotactic protein-1 (MCP-1)^10^, suggesting that AD might participate in the local chronic inflammation and bone erosion in RA. Emerging evidence reveals that AD could increase the migration of synovial fibroblasts (SFs) and lymphocytes in RA^11^, indicating a role of AD involved in the synovitis of RA. Interestingly, recent clinical studies have suggested that serum levels of AD levels are linked to the radiographic progression in RA^12^, implying that AD might participate in bone erosion in RA.

T cells that preferentially produce IL-17 are named T helper cell 17 (Th17 cell), which have been shown to play a key role in orchestrating inflammatory response. In patients with RA, secretion of IL-17 by Th17 cell was found to induce IL-6 in cultured synoviocytes^13^. Besides the production of matrix proteinases, IL-17 can also destroy extracellular matrix and cause bone resorption^14^. In addition, IL-17 could stimulate osteoblasts to express the receptor activator of nuclear factor-κB (RANK) ligand (RANKL), which are critically involved in osteoporosis and bone erosion^15^.

Osteoclasts, formed by fusion of mononuclear precursors of the monocyte/macrophage, are the cell type ultimately responsible for bone destruction in RA. The development, activity, and survival of osteoclasts require an essential osteoclastogenic mediator: RANKL^16^. RANKL, a membrane-residing protein on osteoblasts, interacts with RANK, inducing marrow macrophages differentiation into osteoclasts^17^. RANKL exerts its functions by binding to its unique receptor RANK, and osteoprotegerin (OPG) acts as its natural decoy receptor by blocking the RANK/RANKL interaction. Mice lacking OPG exhibit severe osteoporosis and bone erosion, implicating the importance of RANKL/OPG balance for maintaining osteoclast homeostasis^18^.

Recent studies including our findings have indicated that AD is involved in bone erosion in RA. However, the underlying mechanisms remain unclear. The strong link between RANKL, IL-17 and bone erosion prompted us to examine whether AD could affect Th17 differentiation and RANKL expression and thereby accelerate bone erosion in RA. In the present study, we determined the effect of AD on modulating Th17 cell response and RANKL expression *in vitro* and in mice suffering from collagen-induced arthritis (CIA). We revealed a previously unrecognized role of AD in RA in current study.

## Results

### Local AD, IL-17 and RANKL levels are increased during CIA development

**Figure 1:**
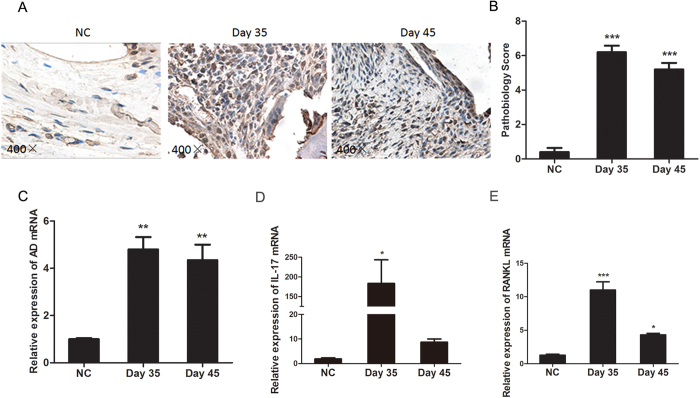
Local AD levels are increased during CIA development. (**A,B**). Representative images of immunohistochemical staining of AD in the joint tissue of DBA controls and CIA mice on day 35 and day 45 post the 1^st^ immunization (n = 3). AD-expressing cells were stained with intense brown color (magnifications: 400 ×). (**C–E**). Relative expression of AD, IL-17 and RANKL mRNA in joint tissues of normal control (NC) and CIA mice were detected by real-time PCR on both day 35 and day 45 post the 1^st^ immunization (n = 7). All data were shown as mean ± SEM (*p < 0.05, **p < 0.01, ***p < 0.001). Results are derived from 3 separate experiments.

To determine the role of AD in the pathogenesis of autoimmune arthritis, we first examined the expression of AD in joint tissue from CIA mice. Joint tissues from DBA/1J and CIA mice on both day 35 (at the early phase of arthritis) and day 45 (at peak phase of arthritis) post the 1^st^ immunization were analyzed by immunohistochemical staining for AD expression. As shown in Fig. 1A-B, the number of AD positive cells was substantially increased in the inflamed joint tissue of CIA mice on both day 35 and day 45. Consistent with the results of immunohistochemical staining, markedly elevated levels of AD transcripts were detected in joint tissue of CIA mice by real-time polymerase chain reaction (PCR) analysis when compared with control mice (Fig. 1C). Moreover, the expression of both IL-17 and RANKL were significantly higher on day 35 than day 45 post the 1^st^ immunization during CIA development (Fig. 1D,E). These data implied a positive correlation of locally increased AD with IL-17 and RANKL expression during CIA progression.

### Local AD injection aggravates CIA mice arthritic development and bone erosion

**Figure 2:**
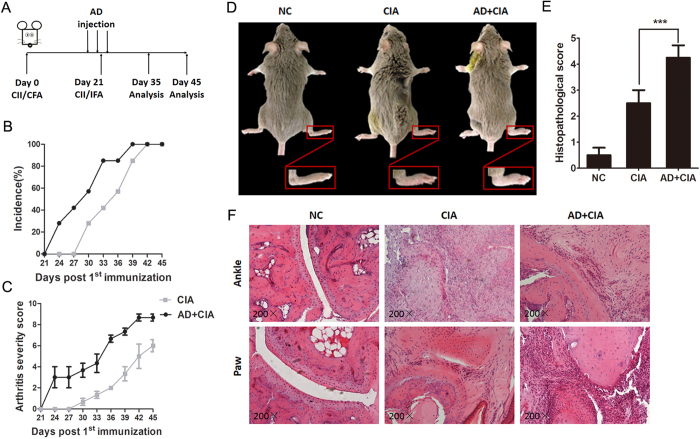
Local AD injection exacerbates CIA development. (**A**). Timetable of CIA induction and AD administration in CII-immunized DBA/1J mice. AD-treated group: CIA mice were injected AD (10 μg/10 μl) on day 17, day 20 and day 23. PBS-treated group: CIA mice were injected PBS (10 μl) on day 17, day 20 and day 23. All mice were sacrificed on day 35 and 45 post 1^st^ CII immunization. (**B,C**). Arthritis severity scores and incidence of CIA development in CIA mice were recorded daily after 2^nd^ CII immunization (n = 7). (**D**). Representative photographs of PBS- and AD treated-CIA mice. (**E**). Values of histopathological scores were shown as mean ± SEM derived from 7 mice per group. (**F**). H&E staining analysis of ankles and paws from AD-treated CIA and PBS-treated controls (magnifications: 200×). All data were shown as mean ± SEM (***p < 0.001). Results are derived from 3 separate experiments.

**Figure 3:**
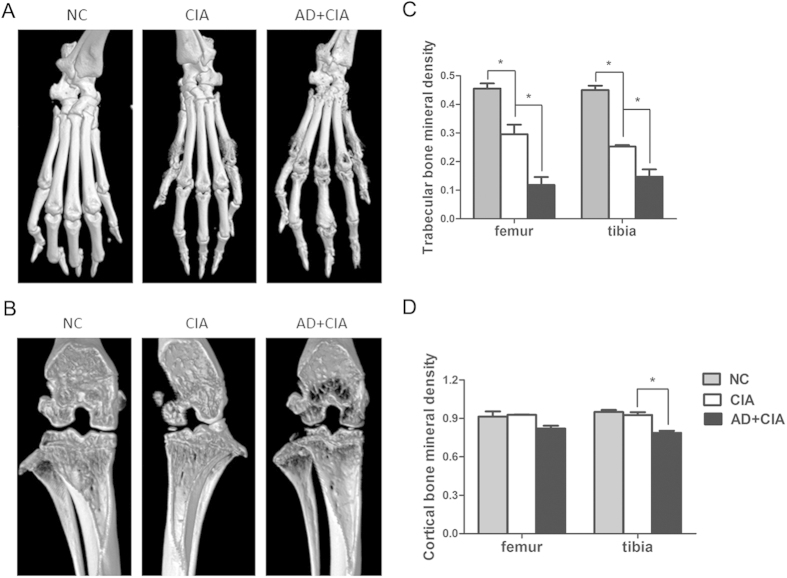
Local AD injection accelerates bone resorbing in CIA. (**A**). Representative three-dimensional renditions of the ankles and paws scanned by Micro-CT. (**B**). Representative three-dimensional renditions of the trabecular bone in NC, PBS-treated and AD-treated CIA were scanned by Micro CT. (**C,D**). Both trabecular and cortical bone mineral density in femur and tibia were calculated. All data were shown as mean ± SEM (*p < 0.05). Results are derived from 3 separate experiments. (n = 3).

Next, we examined whether AD could enhance CIA progression and bone erosion by local injection of AD into the knee joint in mice with CIA. A dose of 10 μg AD was intraarticularly injected into the knee joint of CIA mice consecutively on days 17, 20 and 23 post the 1^st^ type II collagen (CII) immunization of DBA/1J mice (Fig. 2A). As shown in Fig. B–D, AD-treated CIA mice exhibited an earlier onset of arthritis and higher arthritis scores than the control CIA mice treated with phosphate buffered saline (PBS). Histopathological analysis indicated that more pronounced synovial hyperplasia, cartilage damage and bone erosion were observed in AD-treated CIA mice compared to the control CIA mice (Fig. 2E,F). Micro computed tomography (Micro-CT) examination showed that more severe bone erosion was detected in the periarticular bone of paws and ankles from AD-treated CIA mice (Fig. 3A). Moreover, AD-treated CIA mice showed statistically significant decrease in trabecular number and trabecular bone mineral density (BMD) in both femur and tibia compared with PBS-treated group (Fig. 3B,C). The cortical bone mineral density in the tibia was also reduced in AD-treated CIA mice compared to PBS-treated CIA mice, but did not reach statistical significance (Fig. 3D). Taken together, the locally increased levels of AD in the joint of CIA mice could directly exacerbate the arthritis signs, synovial inflammation and joint damage in CIA mice, suggesting that AD might participate in disease progression in CIA mice.

### Increased Th17 cells and its relative cytokines in the joint tissue of AD-treated CIA mice

**Figure 4:**
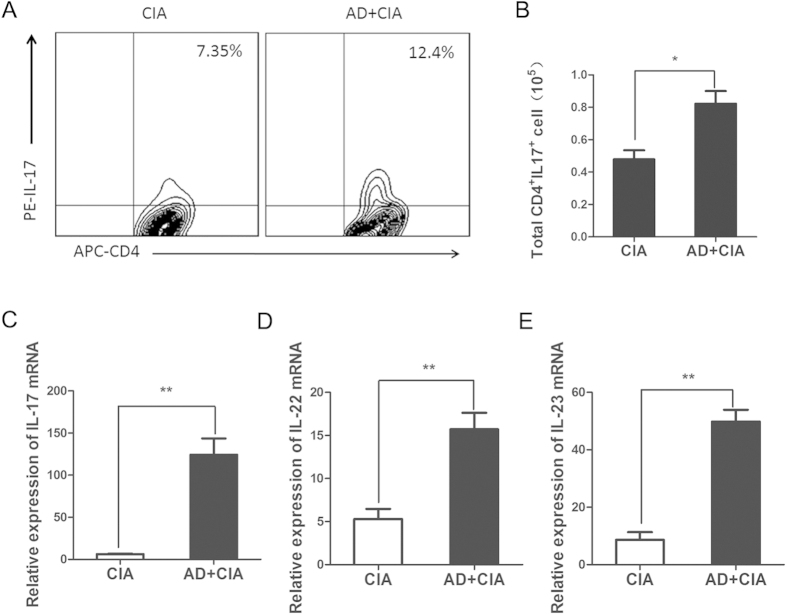
Local AD injection increases Th17 and its transcription factors in joint tissue of CIA (**A,B**). The frequencies and total number of Th17 were analyzed by flow cytomtry on day 45 post the 1^st^ immunization (n = 3). (**C**). Relative expression of IL-17 in joint tissues was determined by real-time PCR on day 45 post the 1^st^ immunization (n = 7). (**D,E**). Relative IL-22 and IL-23 mRNA levels in joint tissue of AD-treated and PBS-treated CIA mice on day 45 post the 1^st^ immunization were measured by real-time PCR analysis (n = 7). All data were shown as mean ± SEM (*p < 0.05, **p < 0.01). Results are derived from 3 separate experiments.

Since IL-17 plays a critical role in bone erosion in RA patient and murine arthritis, we sought to determine whether AD could affect IL-17 and Th17 cells expression in the joint tissue of CIA mice. Flow cytometric analysis indicated CD4^+^IL-17^+^ Th17 cell was an approximate 1.5-fold expansion in joint tissue of AD-treated CIA mice at day 45 (Fig. 4A,B) compare to untreated CIA mice. Consistent with the flow cytometric data, real-time PCR analysis revealed that IL-17 expression showed a 10-fold increase on day 45 post the 1^st^ immunization (Fig. 4C). We also analyzed the expression of IL-17 related factors including IL-22 and IL-23 messenger ribonucleic acid (mRNA) in joint tissues by real-time PCR. The expression of IL-22 and IL-23 in joint tissue were significantly up-regulated in CIA mice after AD injection (Fig. 4D,E). Collectively, these results indicated that AD might be involved in promoting Th17-mediated bone erosion.

### Effect of AD on RANKL expression in joint tissue of CIA mice

**Figure 5:**
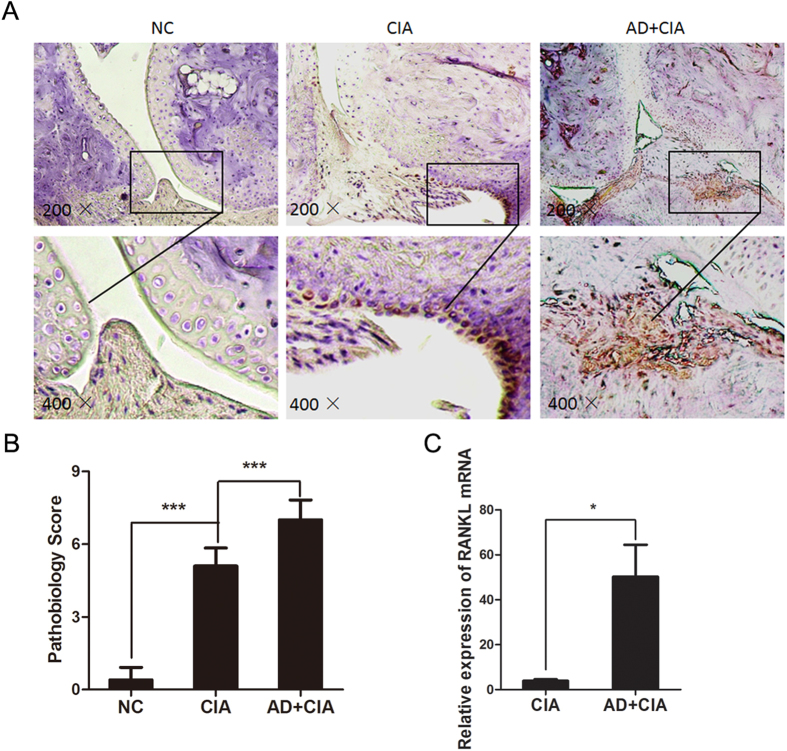
Local AD injection increases RANKL expression in joint tissue of CIA. (**A,B**). Immunohistochemical staining of RANKL in joint tissue of AD-treated and PBS-treated CIA mice on day 45 post the 1^st^ immunization (magnifications: 200 × and 400 ×). RANKL-expressing cells were stained with intense brown color (n = 3). (**C**). Relative RANKL mRNA levels in joint tissue of AD-treated and PBS-treated CIA mice on day 45 post the 1^st^ immunization (n = 7). All data were shown as mean ± SEM (*p < 0.05, ***p < 0.001). Results are derived from 3 separate experiments.

Furthermore, we evaluated potential effect of AD on the expression of RANKL in CIA model. It was found that the expression levels of RANKL reached a peak level on day 45 post the 1^st^ immunization in AD treated mice (Fig. 5C). Immunohistochemical analysis indicated a markedly elevated number of RANKL-expressing cells in the synovium of AD treated mice (Fig. 5A,B). Given most RANKL^+^ cells appear to be synoviocytes, we then treated rheumatoid arthritis synovial fibroblasts (RASFs) with AD. A significantly increased levels of RANKL expression were found in AD-treated RASFs, as compared to untreated RASFs (Supplementary Fig 1. S1). Taken together, our data indicated a direct effect of AD on inducing RANKL expression.

### AD promotes the differentiation of naïve T cells into Th17 cells *in vitro*

**Figure 6:**
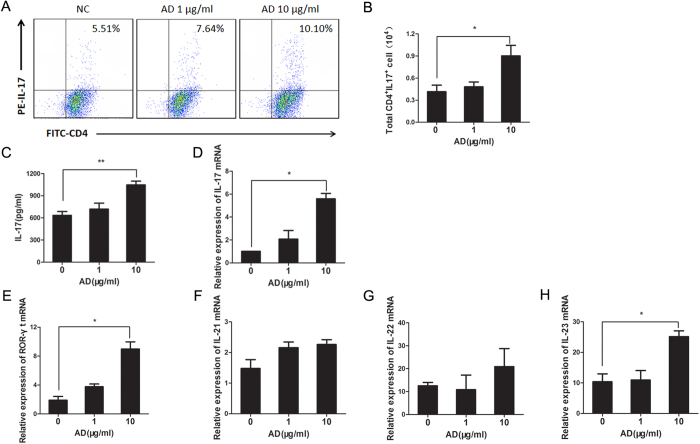
AD promotes Th17 cell differentiation in culture. (**A**). Frequencies of Th17 cells were estimated by flow cytometry. Naïve T cells were cultured under Th17 polarization condition in the absence or presence of different concentrations of AD (1 and 10 μg/ml) for 3 days. (**B**). Total number of Th17 cells from T cell culture with different treatments were counted. (**C**). IL-17 mRNA level was measured by real-time PCR. (**D**). Supernatants were collected for IL-17 detection by ELISA. (**E–H**). Relative ROR-γt, IL-21, IL-22 and IL-23 mRNA levels in naïve T cells cultured with AD treatment under the Th17 polarized condition for 72 hours were measured by real-time PCR. All data were shown as mean ± SEM (*p < 0.05, **p < 0.01). Results are derived from 3 separate experiments. (n = 3)

To further determine whether AD exerts a direct effect on promoting Th17 cell differentiation, naïve CD4^+^ T cells from spleen of C57BL/6J mice were induced towards to Th17 differentiation in the presence or absence of AD for 3 days. The expression of both AdipoR1 and AdipoR2 transcripts could be detected in naïve CD4^+^ T cells and *in vitro*-generated Th17 cells (Supplementary Fig 2. S2). Then, as shown in Fig. 6A,B, the frequencies of Th17 cells were significantly increased in a dose-dependent manner upon AD stimulation (Fig. 6A,B). The mRNA and protein levels of IL-17 in cultured cells and supernatants were also markedly elevated in AD treated T cells (Fig. 6C,D). The levels of retinoid-related orphan nuclear receptor-γt (ROR-γt), IL-21, IL-22 and IL-23 mRNA were determined by real-time PCR when naïve T cells were cultured in the presence of different concentrations of AD for 72 hours. As indicated in Fig. 6E,H, the expression of ROR-γt and IL-23 mRNA were enhanced in a dose dependent manner; however, we failed to detect the changes of IL-21 and IL-22 production *in vitro* (Fig. 6F,G). Together, these results demonstrated that AD could promote Th17 differentiation *in vitro*.

## Discussion

RA is a systemic inflammatory disease that involves a hyperplasia of synovial tissues and a structural damage of cartilage and bone. In the present study, we first found that local AD, IL-17 and RANKL levels are increased during CIA development. Given IL-17 and RANKL are well established as the critical cytokines in mediating bone erosion in RA, here, we addressed whether AD could accelerate bone erosion in RA by prompting Th17 differentiation and enhancing RANKL expression. We have shown that intraarticular injection of AD into the knee joint of CIA can aggravate arthritic progression and bone erosion, which is accompanied by significantly increased number of Th17 cells and a high expression of RANKL in joint tissues. Furthermore, we have identified a previously unrecognized new role of AD in promoting the differentiation of naïve T cells into Th17 cells *in vitro*. Together, our results have demonstrated a critical role of AD in the pathogenesis of autoimmune arthritis.

The synovial tissue is recognized to be actively involved in governing the persistence of inflammatory disease^19^. Cytokine production, particularly IL-17 that arises from synovial tissue, is central to the pathogenesis of bone erosion. IL-17, the major cytokine of Th17 cell, is an important inflammatory cytokine that induces production of prostaglandins, nitric oxide, cytokines and chemokines^5^,^20^,^21^. IL-17 induces the production of IL-1 and TNF in macrophages and fibroblasts and is synergistic with IL-1 in the upregulation of inflammatory mediators released by synovial fibroblasts^22^,^23^. At the same time, IL-17 blocks the function of regulatory T cell (Treg) and Th2 cell, and thereby inhibits local bone erosion as well as systemic bone loss in CIA model of TNF-mediated arthritis^24^, highlighting a critical role of IL-17 in RA pathogenesis^25^. We find that the local increase of AD concentration can enhance the expression of Th17 and its transcription factor in joint tissue. Level of IL-17 mRNA is higher in AD-treated CIA mice especially on day 45 (a 10-fold increase). Moreover, we have also observed increased number of Th17 cells in joint tissues on day 45. These data have allowed us to propose that higher concentration of AD in inflamed joint is much more important in persistence of inflammatory microenvironment than occurrence of acute inflammatory responses.

Our data have clearly shown that AD can promote Th17 cell differentiation through upregulating of the expression of ROR-γt, IL-22 and IL-23 expression. It is currently clear that ROR-γt is a unique transcription factor of Th17 by inducing the transcription of IL-17 gene in naive helper T cells^26^. IL-22 is a cytokine that can induce epithelialcell proliferation and of antimicrobial proteins in keratinocytes^25^. IL-23 is more important for Th17 expansion and stabilization^27^. We have further defined a novel function of AD in promoting Th17 differentiation *in vitro*. Flow cytometry analysis revealed that the frequencies of Th17 cells were significantly increased with the stimulation of AD at a concentration of 10 μg/ml. Notably, the expression of ROR-γt and IL-23 are up-regulated in a dose dependent manner. These results show that a higher expression of AD in inflamed joint is involved in modulating Th17 responses. These results indicate a novel role of AD in promoting Th17 cell differentiation. The biologic effect of AD is mediated through AdipoR that has been validated in previous studies. Further studies are needed to explore how AD acts on AdipoR to promote Th17 differentiation.

Remarkable progress has been made in recent years in the field of osteoclast research primarily due to the finding of the RANKL/RANK system. In the pathological condition of RA, proinflammatory cytokines produced by synovial fibroblasts in the inflamed joints can cause increased local joint RANKL expression^28^. RANKL can stimulate osteoclast differentiation from hematopoietic precursor cells *in vitro*. It can also act on mature osteoclasts and activate the bone-resorbing activity and survival of the cells. RANKL binds to its receptor RANK, which induces intracellular signals including nuclear factor-κB (NF-κB) activation and c-Jun N-terminus kinase activation^29^,^30^. The other important actor in this system is OPG, a soluble receptor of RANKL, which specifically binds to RANKL and inhibits RANKL activity by preventing its binding to RANK^31^. In the present study, we find that the expression of RANKL in AD-treated CIA mice is significantly higher than that in PBS-treated CIA controls. We further observed significantly increased levels of RANKL expression in AD treated RASFs indicating a direct effect of AD on inducing RANKL expression. We suggest that this type of regulation by AD has three protential mechanisms. First, it is possible that AD contributes to enhance the high expression of RANKL directly. In addition, the immunomodulatory role of AD might be another explanation for the regulation of RANKL in RA and arthritis models. Moreover, we have observed that local increase in AD directly modulated Th17 differentiation is accompanied by an increase of the production of IL-17 and IL-22, which are involved in regulating RANKL expression^32^,^33^. These results demonstrate that AD is participated in the regulation of RANK/RANKL/OPG signaling pathway, promoting inflammation-induced osteoclastogenesis in CIA models and RA patients. Further studies are needed to explore whether AD acts to promote inflammation-induced osteoclast activation and bone erosion.

Study by Lee SW *et al.* has shown that AD could mitigate disease severity of CIA model^34^. The data are conflicted with our results. In our experiments, globular AD was used to inject into the knee joint of CIA model; however, full-length AD was used in Lee’s study, which might be an impossible explanation of the opposite effects of AD on CIA between our study and Lee’s. There are mainly two isoforms of AD have been identified in circulation, with globular AD and full-length AD. A recent study by Klaus W Frommer et al have shown that different AD isoforms may induce diverging effects^35^. After globular AD treatment, RASFs showed significantly increased expression of chemokines, proinflammatory cytokines and matrix metalloproteinases (MMPs), suggesting that globular AD might play a more potent proinflammatory role in RA pathogenesis as compared with full-length AD. Due to the intricate interactions of various immune cell types and complicated AD signal pathways involved in disease pathophysiology, further studies are need to determine the exact function of AD at the different stages of arthritis development in mice and RA patients.

Interestingly, Piccio L *et al.*^36^ have revealed that AD deficient mice could develop worse experimental autoimmune encephalomyelitis (EAE) with greater central nervus system (CNS) inflammation, more lymphocytes proliferation, higher amounts of interferon-γ (IFN-γ), IL-17, TNF-α and IL-6 than wild type (WT) mice. However, our data provide the solid evidence that the locally increased AD levels contributed to inflammation and bone erosion in CIA mice by enhancing Th17 response and prompting RANKL expression. These contrasting results may suggest that systemic function and localized effect of AD in autoimmune pathogenesis might be different.

Substantial evidence has suggested that other adipokines, such as leptin and resistin are also involved in regulating disease progression of RA. Deng *et al.* have shown a function of leptin in enhancing Th17 cell response and exacerbating joint inflammation in CIA mice^37^. In addition, resistin levels are found to be higher in the serum and synovial fluid of RA patients than in those with osteoarthritis (OA). The observed statistically significant correlation between synovial fluid resistin levels and rheumatoid factor (RF), anti-citrufllinated protein/peptide autoantibody (ACPA) and Larsen score which further indicate that locally produced adipokines are closely implicated in synovial inflammation during the pathogenesis of RA^38^.

In summary, our studies have demonstrated that AD can promote the differentiation of naïve T cell to Th17 cell and upregulate RANKL/OPG ratio, resulting in an enhanced synovitis and bone erosion in CIA models. These findings reveal a novel role of AD in mediating the development of autoimmune arthritis in mice. More clinical studies are needed to further confirm the potential therapeutic effect of targeting AD in the treatment of RA.

## Methods

### Ethics statement

All experiments were conducted in compliance with the guidelines for the care and use of laboratory animals and approved by Institutional Animal Care and Use Committee of Nanjing Medical University (Permit Number: IACUC-2013090101).

### Experimental animals

Eight- to twelve-week-old DBA/1J mice were purchased from the Shanghai Laboratory Animal Center. Mice were fed under pathogen-free conditions at experimental animal center of Nanjing Medical University. All experiments were conducted according to the animal care and use committee guidelines.

### CIA induction and AD treatment

CIA mice were induced as previously described^39^. Firstly, 100 μg of bovine type II collagen (CII) (Chondrex) was dissolved in 0.05 M acetic acid with an equal volume of Freund’s complete adjuvant (Difco). Then DBA/1J mice were intradermally administered at the base of tail. On Day 21, booster injections were administered with 75 μg of type II collagen and Freund’s incomplete adjuvant (Difco) near the primary injection site. CIA mice were intraarticularly injected with AD (10 μg AD in 10 μl PBS) into knee joints on day 17, day 20 and day 23 post first CII-immunization. Other CIA mice were treated with same volume of PBS as controls. CIA mice were scored for joint inflammation every day post 2^nd^ CII-immunization, with a maximum arthritis severity score of 16 per mouse. Severity of disease was evaluated by visual inspection of the paws. Each paw was scored for the degree of inflammation on a scale from 0 to 4: 0, no evidence of erythema and swelling; 1, erythema and mild swelling confined to the midfoot (tarsals) or ankle joint; 2, erythema and mild swelling extending from ankle to the midfoot; 3, erythema and mild swelling extending from ankle to metatarsal joints; 4, erythema and severe swelling encompassing the ankle, foot, and digits. Scores from all four paws were added to give the total for each animal.

### Cell culture

Purified naïve T cells (CD4^+^CD62L^+^ T cells) from C57 BL/6 mice were isolated by CD4^+^CD62L^+^ T Cell Isolation Kit II (Miltenyi) according to the manufacturer’s instructions and the purity was >94%. Naïve T cells were cultured in 10 μg/ml of anti-CD3 mAb and 3 μg/ml of anti-CD28 mAb precoated in 24-well plates, and then 30 ng/ml of IL-6 (R&D Systems), 30 ng/ml of IL-23 (R&D Systems) and 3 ng/ml of TGF-β (R&D Systems) were added into the culture system in the presence of AD (1 and 10 μg/ml) (R&D Systems) or without AD for 72 hours to induce Th17 differentiation. It had been confirmed that the endotoxin level of AD is <1.0 EU per 1 μg of the protein by the limulus amebocyte lysate (LAL) method.

### Flow cytometric analysis

Single-cell suspensions from mouse spleen and joint tissues were prepared. All samples were treated according to the manufacturer’s recommendations. Anti-CD4, anti-CD3 and anti-IL-17 were used for Th17 cell surface markers staining, they were purchased from BioLegend. In order to perform intracellular staining for IL-17, phorbol myristate acetate (PMA), ionomycin and brefeldin (BFA) (Sigma) were added into the culture system for 5 hours before analysis.

### RNA extraction and real-time PCR analysis

Cells and joint tissues were collected for real-time PCR analysis. RNA samples were extracted by Trizol reagent (Invitrogen) and then RNA was converted to complementary deoxyribonucleic acid (cDNA) using Prime Script^TM^ RT regent Kit according to the manufacturer’s instructions (Takara). PCR primers used for real-time PCR were as follows: for IL-17, sense 5’-CCTCACACGAGGCACAAGTG-3’, antisense 5’- CTCTCCCTGGACTCATGTTTGC-3’; for ROR-γt, sense 5’- CACGGCCCTGGTTCTCAT-3’, antisense 5’- GCAGATGTTCCACTCTCCTCTTCT-3’; for IL-21, sense 5’- TGCTAGCTCCAGCCTCAGTCT-3’, antisense 5’- TTAAGTGCTGAACTTGTTTGGATTG-3’; for IL-22, sense 5’- TCGTCAACCGCACCTTTATG-3’, antisense 5’- CCCGATGAGCCGGACAT-3’; for IL-23, sense 5’- CCTTCTCCGTTCCAAGATCCT-3’, antisense 5’- ACTAAGGGCTCAGTCAGAGTTGCT-3’; for AD, sense 5’- GGAGTGTTCGTGGGCTTAGG-3’, antisense 5’- GCAGCTCCGGTGATATAGAGG-3’; for RANKL, sense 5’-CAGCATCGCTCTGTTCCTGTA-3’, antisence 5’-CTGCGTTTTCATGGAGTCTCA--3’; for β-actin, sense 5’- TGTCCACCTTCCAGCAGATGT-3’, antisense 5’- AGCTCAGTA ACAGTCCGCCTAG-3’. The real-time PCR analysis was detected by an ABI 7900 system (Applied BioSystems Inc) and the cycling parameters were as follows: 95 °C for 10 minutes, followed by 40 cycles at 95 °C for 15 seconds and 60 °C for 1 minute. Relative expression of target genes were calculated as 2^–ΔΔCt^.

### Enzyme linked immune sorbent assay (ELISA)

Levels of IL-17 in the culture supernatants were measured by ELISA (R&D Systems) according to the manufacturer’s recommendations. Briefly, serum samples (1: 5 dilution) and standards were added to the 96-well plates. After incubation for 2 hours and washing 5 times, mouse IL-17 conjugate was added, followed by incubation with TMB substrate solution and stop solution. The intensity of the color reaction was measured by a microplate reader at a wavelength of 450 nm. Concentrations of IL-17 were determined by a standard curve according to the manufacturer’s instructions.

### Haematoxylin & eosin (H&E) staining and immunohistochemical analysis

Paw and knee joints of CIA mice were removed from sacrificed mice for H&E staining and immunohistochemical analysis. Samples from each group were fixed in 4% buffered paraformaldehyde, decalcified in 50 mM ethylene diamine tetraacetic acid (EDTA). Then tissues were sectioned to 3 μm thickness, deparaffinized in xylene and rehydrated through a series of concentrations of ethanol. The sections were prepared sagittal and stained with H&E and after inactivation of endogenous peroxidase, sections were blocked by incubation with 5% bovine serum album for 30 minutes at room temperature, then incubated with rabbit anti-human RANKL antibody (Abcam) diluted 1:50 at 4 °C overnight in a humidified chamber. After washing, sections were next incubated with peroxidase-conjugated goat anti-rabbit secondary antibody for 1 hour at room temperature. The reactions were developed using a DAB substrate kit, with hematoxylin as counterstain. Each slide was evaluated by one of the authors under a microscope (Nikon). Tissue sections were scored for staining of the lining on a 0 to 5 scale as follows: 0, no staining; 1, few of the cells positively stained; 2, some (fewer than half) of the cells stained; 3, approximately half of the cells stained; 4, more than half of the cells stained; and 5, all cells stained. For each section, the number of positively stained cells was counted in 20 fields.

### Micro-CT

Computed tomographic images of the knee joints and paws of the mice in all three groups (n = 3) were acquired on day 45 post the 1^st^ immunization, using a Micro-CT Scan SkyScan1176S scanner at a resolution of 9 μm. For verification of bone destruction, 3-dimensional models of the knee joints and paws were reconstructed using SkyScan CT Analyzer version 1.8. BMD were assessed by scanner software (Skyscan CTAn).

### Statistical analysis

Data are expressed as the mean ± standard error of the mean (SEM). Statistical analysis was performed using one-way analysis of variance (ANOVA) tests. Results are derived from 3 separate experiments. The values of p < 0.05 were considered significant.

## Additional Information

**How to cite this article**: Sun, X. *et al.* Adiponectin exacerbates collagen-induced arthritis via enhancing Th17 response and prompting RANKL expression. *Sci. Rep.* **5**, 11296; doi: 10.1038/srep11296 (2015).

## Supplementary Material

Supplementary Information
