# Supplementary material for: Adiponectin exacerbates collagen-induced arthritis via enhancing Th17 response and prompting RANKL expression
Source: Sci Rep. 2015 Jun 11;5:11296. doi: 10.1038/srep11296 (PMC4462752; doi:10.1038/srep11296)
Supplement: Supplementary Information [file srep11296-s1.doc]

**Adiponectin exacerbates collagen-induced arthritis via enhancing Th17 response and prompting RANKL expression**

Xiaoxuan Sun^1^, Xiaoke Feng^1, 2^, Wenfeng Tan^1^, Na Lin^1^, Minhui Hua^1^, Yu Wei^1^, Fang Wang^3^, Ningli Li^4^, Miaojia Zhang^1*^

^1^Department of Rheumatology and Immunology, ^2^Department of Traditional Chinese Medicine, ^3^Department of Cardiology. The First Affiliated Hospital of Nanjing Medical University, Jiangsu, China.

^4^Shanghai Institute of Immunology. Institute of Medical Sciences, Shanghai Jiao Tong University School of Medicine, Shanghai, China.

***Correspondence:** Miaojia Zhang, MD.

E-mail: [miaojia_zhang@163.com](mailto:miaojia_zhang@163.com)


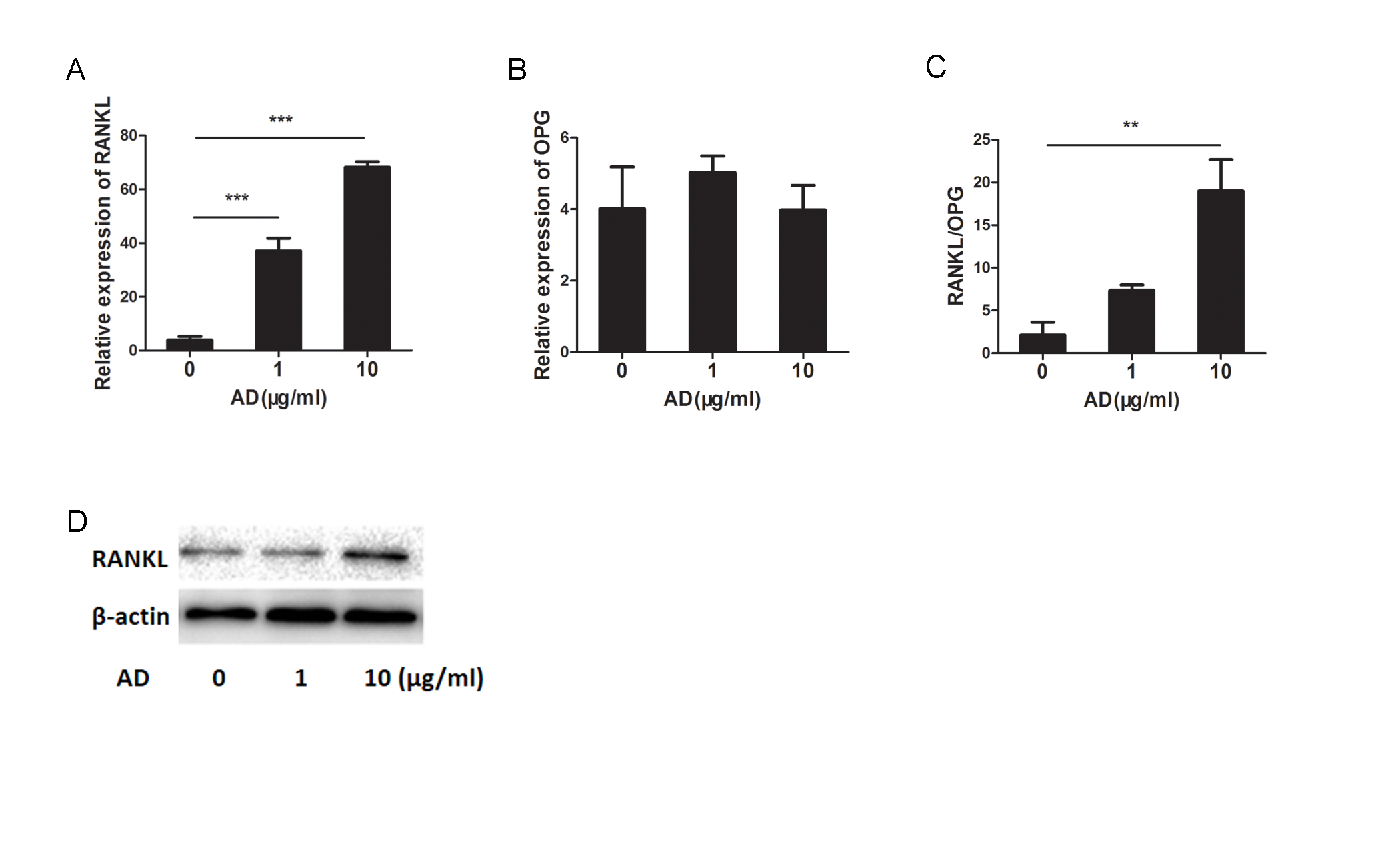


**Supplementary Fig 1. S1. Effect of AD on RANKL and OPG expression in RASFs.**

RASFs were obtained from synovial tissues of 3 RA patients during synovectomy. The RASFs were cultured with AD (0, 1, 10 μg/ml) for 48 h. (A-B). The effects of AD on RANKL and OPG mRNA expression in RASFs were analyzed by real-time PCR. (C). The alteration of RANKL/OPG ratio in RASFs was analyzed. (D). The protein level of RANKL expression in RASFs was tested by western-blot. The data shown are the mean ± SEM for three independent experiments (**p < 0.01, ***p < 0.001). (n=3)

**
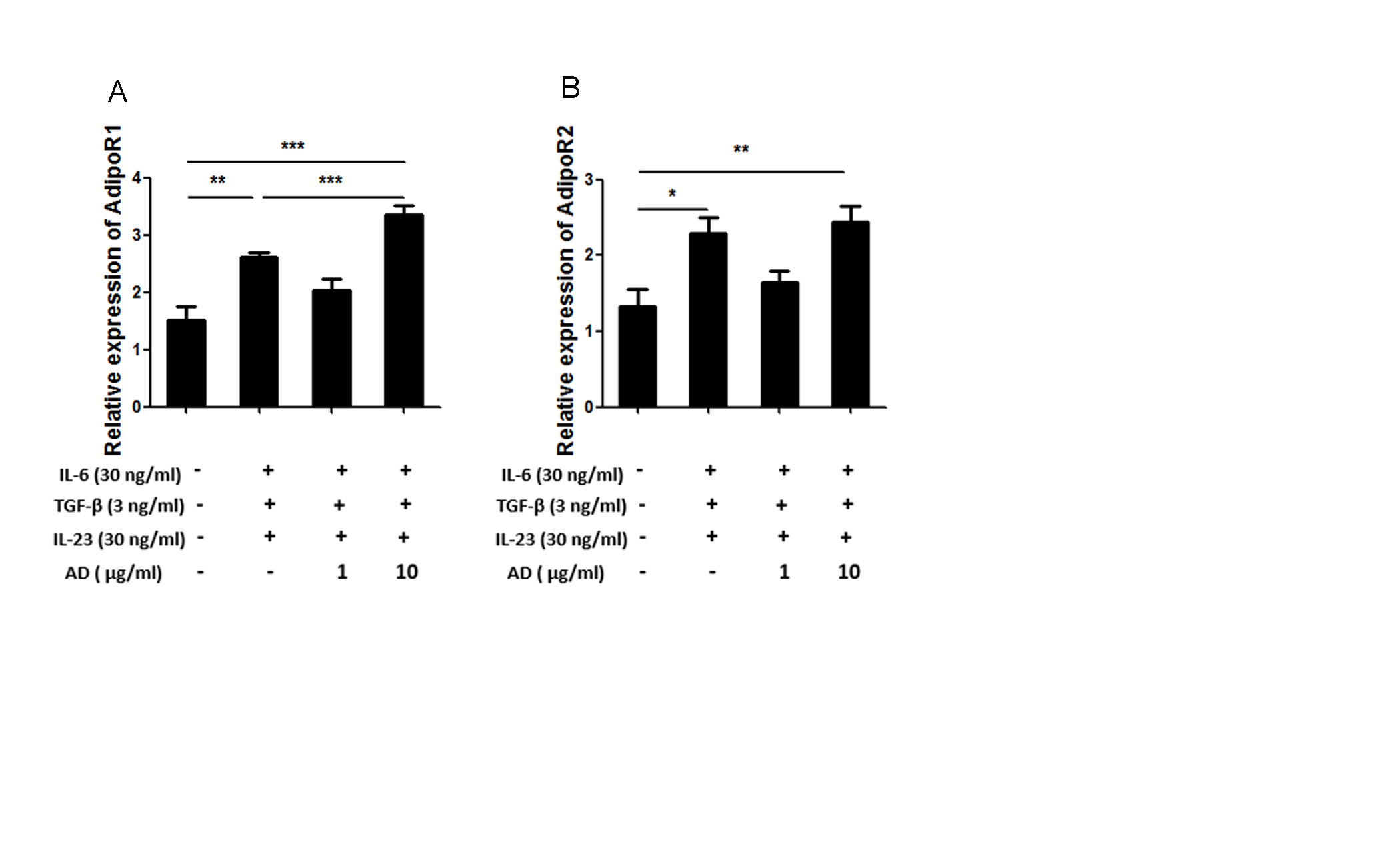
**

**Supplementary Fig 2. S2. Expression of AdipoR transcripts in naïve CD4^+^ T cells and in** **vitro-generated Th17 cells.**

Purified naïve T cells were isolated by CD4^+^CD62L^+^ T Cell Isolation Kit II and the purity was >94%. Naïve T cells were cultured in anti-CD3 mAb and CD28 mAb in 24-well plates overnight, and then were treated with in the presence of AD (1 and 10 μg/ml) or absence of AD for 72 hours to induce Th17 differentiation. (A-B). Expression of AdipoR1 and AdipoR2 mRNA in naïve CD4^+^ T cells and in *vitro*-generated Th17 cells were detected by real-time PCR. The data shown are the mean ± SEM for three independent experiments (*p < 0.05, **p < 0.01, ***p < 0.001). (n=3)


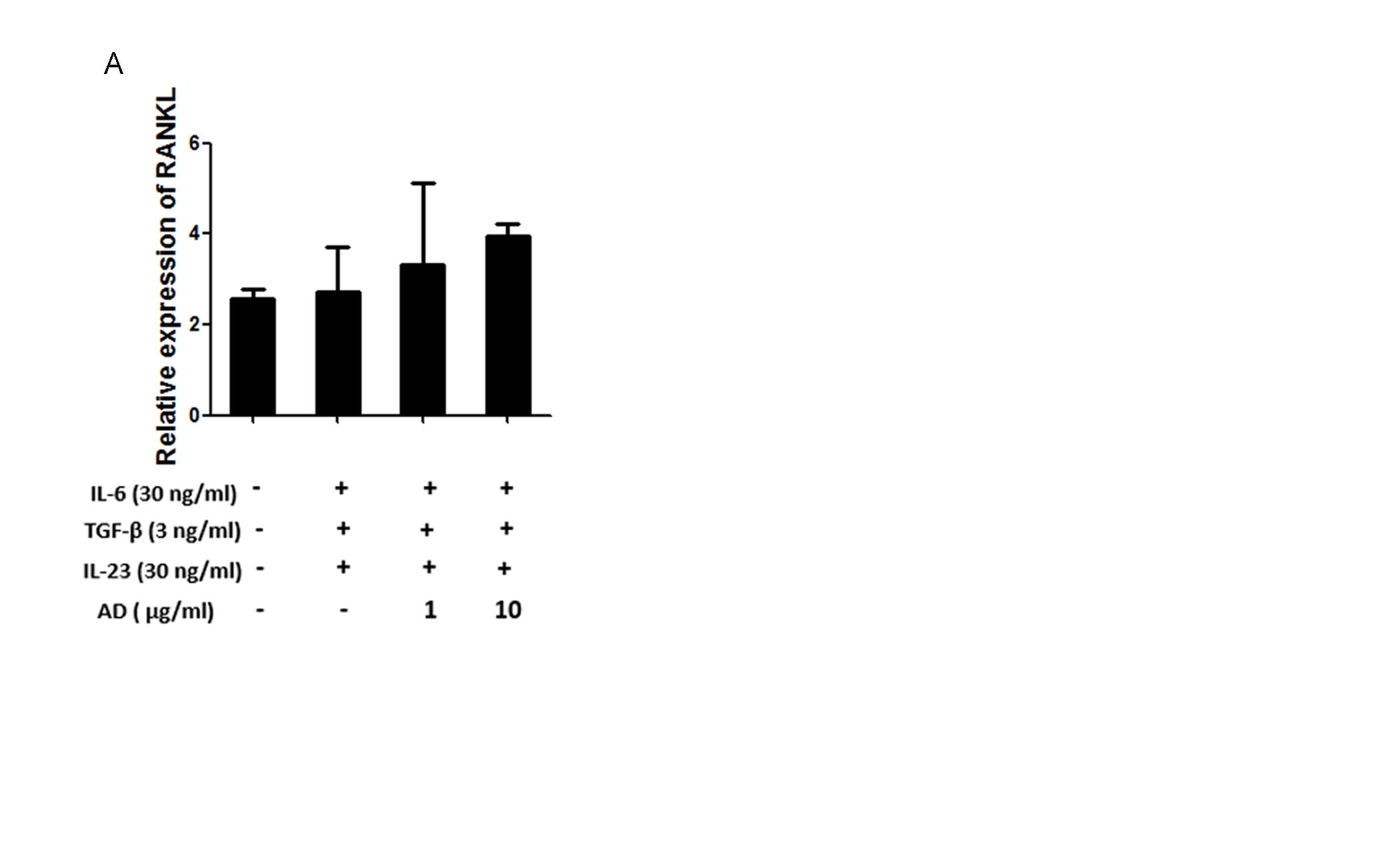


**Supplementary Fig 3. S3. The expression of RANKL transcripts in AD-treated Th17 cells.**

Purified naïve T cells were isolated by CD4^+^CD62L^+^ T Cell Isolation Kit II and the purity was >94%. Naïve T cells were cultured in anti-CD3 mAb and CD28 mAb in 24-well plates overnight, and then IL-6, IL-23 and TGF-β were added into the culture system in the presence of AD (1 and 10 μg/ml) or without AD for 72 hours to induce Th17 differentiation. (A). The expression of RANKL transcripts in AD-treated Th17 cells were detected by real-time PCR. The data shown are the mean ± SEM for three independent experiments. (n=3)
